# Designing strategies to support Implementation of iNtensive Therapy for Early Reach through PLAY (INTERPLAY) for young children with cerebral palsy: a study protocol

**DOI:** 10.1186/s43058-024-00602-y

**Published:** 2024-06-18

**Authors:** Alicia Hilderley, Christine Cassidy, Sandra Reist-Asencio, Chelsea Tao, Stephen Tao, Susan McCoy, Divya Vurrabindi, Kathleen O’Grady, Mia Herrero, Liz Cambridge, Eleanor Leverington, Victoria Micek, John Andersen, Darcy Fehlings, Adam Kirton

**Affiliations:** 1 University of Calgary, 28 Oki Drive NW, Calgary, AB T3B 6A8 Canada; 2 Dalhousie University, 6299 South St, Halifax, NS B3H 4R2 Canada; 3 Alberta Children’s Hospital, 28 Oki Drive NW, Calgary, AB T3B 6A8 Canada; 4 Glenrose Rehabilitation Hospital, 10230 111 Avenue NW, Edmonton, AB T5G 0B7 Canada; 5 Holland Bloorview Kids Rehabilitation Hospital, 150 Kilgour Rd, East York, ON M4G 1R8 Canada; 6 University of Toronto, 27 King’s College Cir, Toronto, ON M5S 1A1 Canada; 7 Alberta Health Services, 31 Sunpark Plaza SE, Calgary, AB T2X 3W5 Canada; 8 University of Alberta, 10230 111 Avenue NW, Edmonton, AB T5G 0B7 Canada

**Keywords:** Cerebral palsy, Early intervention, Implementation science

## Abstract

**Background:**

Intensive manual therapy is important for improving lifelong upper limb motor outcomes for infants and toddlers with cerebral palsy. This play-based therapy is delivered by caregivers who are coached by occupational therapists. However, access to this therapy is very limited for Canadian children with cerebral palsy younger than two years old. This project aims to first identify barriers and facilitators and then design implementation strategies to support early intensive manual therapy delivery for infants and toddlers with cerebral palsy across Canada.

**Methods:**

A mixed-methods sequential explanatory design will be used with four consecutive phases. The updated Consolidated Framework for Implementation Research will guide the study. Quantitative data will be collected from a survey in Phase One. Participants will be recruited from three groups: (1) Caregivers of children with cerebral palsy six years old and younger who are eligible for manual therapy; (2) occupational therapists who treat children with cerebral palsy; and (3) healthcare administrators or people responsible for managing pediatric occupational therapy programs. In Phase Two, quantitative data from the survey will be used to map to implementation strategies known to be effective at addressing the identified modifiable barriers and facilitators. Phase Three will collect qualitative data from semi-structured interviews for the purpose of explaining Phase One quantitative findings in greater depth, and for understanding the appropriateness of strategies identified in Phase Two. The participant recruitment strategy and interview guide content for Phase Three will be informed by results of Phase One. Phase Four will use a modified nominal group technique to refine and prioritize an implementation strategy toolbox. Results will be widely disseminated to knowledge users to provide them with tailorable strategies to increase delivery of early intensive manual interventions.

**Discussion:**

This study will provide a comprehensive understanding of the barriers and facilitators to implementation of early intensive manual therapy for young children with cerebral palsy in Canada. A toolbox of evidence-based and tailorable implementation strategies will be disseminated nationally to support uptake of early intensive manual therapy into clinical practice for young children with cerebral palsy.

Contributions to literature

- This study is the first to assess Canadian barriers and facilitators to early intensive manual therapy for young children with cerebral palsy.
- Identified barriers and facilitators will be mapped to evidence-based implementation strategies.
- A comprehensive understanding of barriers, facilitators, and implementation strategies will be obtained from knowledge user groups using mixed methods.
- A final toolbox of implementation strategies will be disseminated to support increased therapy implementation to improve outcomes for young children with cerebral palsy.

## Background

International clinical best practice guidelines emphasize the need to provide early intervention to children with cerebral palsy (CP) before the age of two years old [1], yet few Canadian sites deliver early interventions. It is unknown why disparities in therapy access exist, which prevents identification and delivery of appropriate implementation strategies to ensure children are offered early intervention. Improving implementation first requires an in-depth understanding of the barriers and facilitators to implementation. Appropriate strategies can then be identified to increase national implementation.

The first two years of life is a critical period of neuroplasticity when intervention may positively alter developmental trajectories and optimize lifelong outcomes [2]. High therapy intensity and dose may relate to improved outcomes [3, 4], often requiring caregivers to be the primary therapy deliverers in a therapist-supervised home-based model. Therapy for young children is play-based, with children engaged in enjoyable activities that elicit movement of the targeted limb. For children with CP who have a hand asymmetry, there are two evidence-based approaches: constraint-induced movement therapy (CIMT) or bimanual therapy [1]. CIMT involves constraining the preferred hand to increase use of the more affected hand, while bimanual therapy focuses on using both hands together. Outcomes from CIMT and bimanual therapy appear to be equivalent [5], and these therapies are often used in combination and referred to collectively as “intensive manual therapy”. Understanding how to improve implementation of early intensive manual therapy is critical to improve hand function and lifelong functioning for young children with CP.

Clinical adoption of evidence-based practices, such as early intensive manual therapy, is typically delayed by years and rarely adopted sustainably. Implementation science provides the necessary methodology to examine barriers to implementation and facilitate successful adoption of evidence-based practices into clinical settings through systematic examination of strategies to support integration and sustainability. Identifying an appropriate framework, model, or theory to guide the implementation study is a first step. The Consolidated Framework for Implementation Research (CFIR) [6, 7] is well suited to the aims of this research study, as it provides a systematic and practical approach to identifying potential barriers and facilitators to intervention implementation. Further, identified barriers and facilitators can be mapped to implementation strategies that are known to be effective at addressing these barriers and facilitators using the Expert Recommendations for Implementing Change (ERIC) taxonomy [8].

The CFIR includes five domains, one being focused on individuals who implement, deliver, and/or receive an intervention and therefore are likely to have knowledge of barriers and facilitators. For early intensive manual therapy, these individuals are caregivers of young children with CP, pediatric occupational therapists, and healthcare administrators with decision making power in pediatric occupational therapy programs. Learning the factors that influence therapy delivery from the perspectives of these key individuals will enable identification of implementation strategies.

The primary aims of this study are:

1. To identify barriers and facilitators to delivery of early intensive manual therapy in Canada from the perspectives of parents/caregivers of children with CP, occupational therapists, and healthcare administrators.
2. To co-design implementation strategies to address the identified barriers and leverage facilitators.

## Methods

A mixed-methods sequential explanatory design will be used with four consecutive phases [9]. The protocol follows the Standards for Reporting Implementation Studies (StaRI checklist) [10].

### Study team and governance

The multidisciplinary research team is composed of three co-principal investigators, an implementation scientist co-investigator, a postdoctoral fellow, a graduate trainee, and caregiver and knowledge user partners. Partners include four caregivers of a child with CP, three pediatric occupational therapists, and two clinical/team leads of pediatric occupational therapy programs. Invitations to join the research team were directly sent to potential caregiver and knowledge user partners by known members of the research team. An invitation flyer for caregivers provided them with basic information about the study and role to inform their decision. An initial meeting with one research team member (AH) was held for the purposes of discussing the study, role, and relationship building. The Strategy for Patient Oriented Research (SPORT) pillars of patient engagement were prioritized: Inclusiveness, Support, Mutual Respect and Co-Build [11].

A terms of reference document was created to outline shared expectations between the researchers and partners on the team. Separate documents were made for each partner group. Annual honoraria (caregivers) and hourly compensation (occupational therapists) were detailed. Caregiver honoraria will be $600 CAD every six months, equivalent to 40 hours per year at $30 per hour. Occupational therapists will track their hours and bill their hourly wage. Compensation will not be provided to leads as this study falls within their professional role. The terms of reference remains a living document that is used guide communication frequency and mode, meetings, document review, and other research team activities. Revisions require approval by all parties.

Caregiver and knowledge user partners were engaged in protocol development using an integrated knowledge translation approach, joining the research team in the early stages of project conception. Partners provided detailed feedback on the first protocol draft, and all agreed to final changes before submission to the study funder. Partners will continue to be engaged in all stages of the research project. Input and guidance will be sought from partners on survey and interview guide development, recruitment strategies, data interpretation, and knowledge mobilization materials. Caregiver partners will also be engaged in data collection as co-interviewers for caregiver participant interviews. All partners will be asked to complete Public and Patient Engagement Evaluation Tool (PPEET) [12] after each study phase. This will provide opportunity for the research team to improve the engagement environment and activities, as needed. The Guidance for Reporting Involvement of Patients and the Public (GRIPP-2) long form [13] will be used to report knowledge user involvement in the research study.

The study was approved by four institutional research ethics boards. Data collection will occur through one central site. Participants will provide informed consent separately for participation in each study phase. Research team partners will provide informed consent for completion of the PPEET.

### Implementation framework

The CFIR [6, 7] will guide this research to identify and describe modifiable factors that are facilitators or barriers to early intensive manual therapy delivery. The updated CFIR [6] is composed of 48 constructs and 19 subconstructs within five major domains: innovation, outer setting, inner setting, individuals, and implementation process.

The CFIR will guide examination of contextual influences to implementation across multiple domains. First, the outer setting domain. As health care is funded and administered differently by each province and territory, sites within each of the 13 Canadian provinces and territories will be included. The influence of the inner setting domain will be examined through inclusion of participants at tertiary sites and rural/community practices. The inner setting will include consideration of the practice context, specifically caregiver vs., therapist-driven practice models. The implementation process domain of CFIR examines influences such as the influence of opinion leaders and external change agents. Demographic questions and questions guided by the Individuals domain of the CFIR will support examination of how knowledge user characteristics influence implementation. Questions selected will be informed by research team partners and the literature, with the following areas identified at outset: primary language, family structure, ethnicity, socioeconomic status, and social supports.

### Participants and setting

Participants will be recruited from three groups: (1) caregivers of children with CP six years old and younger who are eligible for manual therapy (i.e., with a clinician-identified hand asymmetry due to CP); (2) occupational therapists who treat children with CP; and (3) healthcare administrators, team leads, or people responsible for managing pediatric OT programs. Considering any implementation of early intensive hand therapy would have happened in recent years, child age of caregiver participants was limited to six years old.

We will use a stratified purposive sampling strategy with convenience sampling techniques to recruit participants, with minimum one participant from each identified site. Data will be collected from 26 national sites. From each province and territory, we intend to recruit from a minimum of one tertiary site (where available) and one rural or community practice. All research activities will occur virtually, either online or by telephone.

### Phase one data collection and analysis

In Phase One, an online survey with Likert-scale response options will be distributed to caregivers, therapists, and healthcare administrators via email, text message, and/or a social media platform. The survey will be built using Qualtrics^XM^ survey software (Qualtrics, Provo, USA). Relevant constructs will be selected by research partners from the list of CFIR constructs. Survey statements for each selected construct will then be drafted, wording reviewed, and additional questions and/or prompts added as needed. Survey questions and wording will be tailored to each participant group based on input and review by research partners. Positive and negatively worded statements will be included. The Likert scale will have five response options: Strongly Disagree, Disagree, Neutral, Agree, Strongly Agree. Participants will have a sixth response option of “Not Applicable” or equivalent wording for statements that do not apply to them.

Participant demographics, manual therapy access and participation (for caregivers), and therapy delivery history (for occupational therapists) will be queried. The survey will be pilot tested before distribution and automated reminders will be sent to maximize response rates. We will employ strategies to promote equitable and inclusive participation. The caregiver survey version will be available in English, French, and Arabic. Participants can complete the survey directly online or choose to have the survey read to them and then entered anonymously into Qualtrics^XM^ for analysis. Open text box screening questions will probe eligibility and legitimacy. Security features will also be enabled in Qualtrics^XM^ to mitigate the risk of fraudulent and bot replies.

Quantitative survey responses will be analyzed using descriptive statistics. Likert-scale response options will be numerically coded, with scores reversed for negatively worded questions. Response frequency will be used to identify the top five barriers and top five facilitators per group. Demographics and data on early intervention access/delivery will be summarized. Where appropriate, data will be compared for each participant group and any meaningful subgroups following consideration of province, site category (e.g., tertiary centre), or other grouping that emerges (e.g., demographics). Mode response scores of each statement will be compared between subgroups, and differences of ≥ 2 will be reported.

### Phase two data collection and analysis

Phase Two will consist of a mapping exercise completed by the multidisciplinary research team. The most frequently endorsed barriers and facilitators from each group and/or subgroup in Phase One will be reviewed and those that are deemed modifiable by the majority of the research team (i.e., ≥ 50%) will be retained for subsequent steps. The research team will consider team expertise, budget, and timelines when deciding on modifiability.

Implementation strategies will then be mapped to these modifiable barriers and facilitators using the ERIC taxonomy [8], which was developed by asking experts to select the top seven strategies for each CFIR construct. The modifiable barriers and facilitators will be inputted to the RISOME Query Tool [14], which outputs a list of possible strategies and the percent of experts who endorsed the match. The research team will vote on the appropriateness of strategies that ≥ 20% experts endorsed for each barrier and facilitator. The APEASE criteria will be considered when voting (Affordability, Practicability, Effectiveness, Acceptability, Safety, Equity) [15]. All strategies endorsed by the majority of the research team (i.e., ≥ 50%) will be included in subsequent phases. If majority is not met for any of the strategies for a barrier/facilitator, the highest endorsement value will be the criteria to select strategies to ensure that at minimum one strategy per barrier/facilitator is included in subsequent phases.

### Phase three data collection and analysis

In Phase Three, a subset of survey respondents will be invited to complete a semi-structured one-to-one interview via telephone or videoconference. All interviews will be facilitated by a trained team member with caregiver interviews co-facilitated by a trained caregiver partner. Training will involve four one-hour interview training sessions to familiarize interviewers with interviewing best practices and the interview guide, and to conduct practice interviews.

Interviews will be divided into two parts. First, an in-depth exploration of the barriers and facilitators identified in Phase One, including the modifiability of these barriers and facilitators. Second, a discussion of the appropriateness and feasibility of implementation strategies mapped in Phase Two. Interview guides will be created with research team partners. The first part of the interview guide will use the updated CFIR Interview Guide as a starting point. For the second part of the interview guide, strategies selected in Phase Two will be categorized using concept mapping [16]. Interview questions will cover each category. Interpreters will be engaged for interviews as needed. Participation over telephone or in-person administration will be options if high-speed internet access is limited, for example in rural and remote communities. We will continue to reflect and create solutions for disparities that may impact study participation. These will be reported to enhance understanding of inequities.

A directed content analysis approach will be used to systematically categorize the data about barriers and facilitators into CFIR domains, followed by inductive thematic analysis [17, 18]. First, two reviewers will read the interview transcripts and categorize similar statements into CFIR domains using an NVivo software (Lumivero, Denver, USA) project template pre-populated with CFIR construct codes. Then deductive analysis using CFIR codes will be conducted, followed by an inductive analysis of the coded data to generate descriptive themes. Interview data about implementation strategies will also be similarly analyzed, but for these data, coding and subsequent categories will be data-driven. As with Phase One, findings from relevant data groupings will be compared for areas of agreement, partial agreement, silence, or dissonance.

### Phase four data collection and analysis

Phase Four will involve a virtual co-design session with participants who were involved in Phase One or Three. A facilitator will lead a modified nominal group technique (NGT) exercise, which is a systematic procedure to obtain group consensus [19]. Using this methodology, the facilitator will first present an overview of results from previous phases. Participants will then independently and silently brainstorm ideas about the strategies, then will share ideas, followed by a round-robin discussion. The final step is a vote to democratically prioritize refined implementation strategies to ensure the final bundle of strategies is manageable and appropriate for implementation. Participants will rank refined implementation strategies and then sum scores will be calculated. Different rankings for groupings will be completed as needed. We will apply the APEASE criteria during the co-design session to ensure the strategies are acceptable, practical, effective, affordable, safe, and equitable. Interpreters will be engaged as needed.

### Sample size

The Phase One survey has a minimum sample of 78, with ideally one respondent from each participant group from each of the potential 26 sites. This sample size will ensure multiple replies from each province, site category, and participant group. The survey will be distributed to over 130 potential respondents to account for nonresponse.

Invitations for Phase Three interviews will be sent to survey respondents until 30 participants consent. Participants will be randomly selected based on groupings informed by Phase One using a purposive, stratified sampling approach. Snowball sampling techniques will be used if additional participants are needed. Based on similar qualitative studies [20], a sample of 30 participants is expected to be sufficient to achieve data saturation.

Phase Four will include 12 participants from Phase One or Three. Invitations will be sent strategically to ensure the group is representative of previous phases.

## Knowledge mobilization plans

The overall knowledge mobilization (KM) goals of this project are to share identified barriers and facilitators with knowledge users and to provide them with implementation strategies to increase delivery of early intensive manual interventions. An Integrated Knowledge Translation (IKT) approach is being used, such that the knowledge users are involved as meaningful partners throughout the research process. Caregiver partners, occupational therapists, and healthcare administrators on our team will work together and in separate groups, as appropriate, to guide strategies. We will also engage clinician scientists with allied health positions whose mandate includes ITK and KM. While integrated KM will be informed through continued discussions with knowledge users, monthly newsletters will be distributed to the multidisciplinary research team, outlining project updates and upcoming activities. A biannual newsletter will also be distributed to survey respondents to update them on study progress and findings. The goals of the newsletter are to: 1) keep respondents engaged for subsequent study phases; 2) ensure respondents are aware that implementation strategies will be shared; and 3) encourage respondents to be receptive to trialing implementation strategies to increase national delivery of early intensive manual therapy.

End-of-project KM will include an array of resources determined and designed in partnership with knowledge users to ensure their needs and goals are considered. Engaged knowledge users include caregivers, occupational therapists, and healthcare administrators, including those in clinical educator roles. These knowledge user partners will inform all KM strategies, through individual, partner group, and larger research team meetings. KM strategies may involve creating new resources and/or informing or expanding existing resources, such as communities of practice.

While KM outputs for implementation strategies cannot be predicted before study conclusion, certain strategies have been identified at project outset. Survey results, interview results on barriers and facilitators, and implementation strategies will be published in academic journals and presented at scientific and non-scientific conferences and/or meetings. Results will be shared with survey respondents and across social media networks using products and strategies informed by knowledge users. KM outputs for implementation strategies will be determined by the strategies and knowledge users. For example, if educational materials are identified as an implementation strategy, materials will be created and distributed in a manner determined by knowledge users. Note that we are purposefully not adding our hypotheses, as we are aware that our team each has their own opinions and biases and do not want these to influence or direct results.

## Discussion

The INTERPLAY study will provide the necessary information and strategies to support increased implementation of early intensive manual therapy for young Canadian children with CP. By capitalizing on the strengths of quantitative and qualitative methods, this study will provide a more complete and in-depth understanding of barriers and facilitators and implementation strategies. Tailorable implementation strategies will be communicated directly to knowledge users, with a clear acknowledgement that strategies have not been tested and may not be effective. We will highlight that strategies are theory and evidence-informed, as well as co-designed with knowledge users to enhance likelihood of success.

This study protocol provides an example of rigorous use of implementation science in the field of pediatric rehabilitation. Continued integration of implementation science methods in this field is necessary to ensure evidence-based rehabilitation therapies are promptly, successfully, and sustainably translated into standard clinical practice. Accelerating the advancement of clinical practice will ensure children are provided with optimal therapies to realize their potential.

## Data Availability

Not applicable.
